# Top Three Pharmacogenomics and Personalized Medicine Applications at the Nexus of Renal Pathophysiology and Cardiovascular Medicine

**DOI:** 10.2174/187569211798377135

**Published:** 2011-12

**Authors:** Murielle Bochud, Michel Burnier, Idris Guessous

**Affiliations:** 1 Institute of Social and Preventive Medicine, Centre Hospitalier Universitaire Vaudois and University of Lausanne, Lausanne, Switzerland; 2 Service of Nephrology, Centre Hospitalier Universitaire Vaudois and University of Lausanne, Lausanne, Switzerland; 3 Unit of Population Epidemiology, Division of Primary Care medicine, Department of Community Medicine and Primary Care and Emergency Medicine, Geneva University Hospital, Geneva, Switzerland

**Keywords:** ACE inhibitors, blood pressure, chronic kidney disease, pharmacogenomics genes, renal function, salt intake, VDR agonists.

## Abstract

Pharmacogenomics is a field with origins in the study of monogenic variations in drug metabolism in the 1950s. Perhaps because of these historical underpinnings, there has been an intensive investigation of 'hepatic pharmacogenes' such as CYP450s and liver drug metabolism using pharmacogenomics approaches over the past five decades. Surprisingly, kidney pathophysiology, attendant diseases and treatment outcomes have been vastly under-studied and under-theorized despite their central importance in maintenance of health, susceptibility to disease and rational personalized therapeutics. Indeed, chronic kidney disease (CKD) represents an increasing public health burden worldwide, both in developed and developing countries. Patients with CKD suffer from high cardiovascular morbidity and mortality, which is mainly attributable to cardiovascular events before reaching end-stage renal disease. In this paper, we focus our analyses on renal function before end-stage renal disease, as seen through the lens of pharmacogenomics and human genomic variation. We herein synthesize the recent evidence linking selected Very Important Pharmacogenes (VIP) to renal function, blood pressure and salt-sensitivity in humans, and ways in which these insights might inform rational personalized therapeutics. Notably, we highlight and present the rationale for three applications that we consider as important and actionable therapeutic and preventive focus areas in renal pharmacogenomics: 1) ACE inhibitors, as a *confirmed* application, 2) VDR agonists, as a *promising* application, and 3) moderate dietary salt intake, as a *suggested* novel application. Additionally, we emphasize the putative contributions of gene-environment interactions, discuss the implications of these findings to treat and prevent hypertension and CKD. Finally, we conclude with a strategic agenda and vision required to accelerate advances in this under-studied field of renal pharmacogenomics with vast significance for global public health.

## 1 INTRODUCTION

Chronic kidney disease (CKD) represents an increasing public health burden [1]. CKD prevalence in adults varies from 5% to 16% worldwide [2-6]. CKD is highly prevalent in western countries [3] as well as in Asia [7, 8]. Epidemiological data in certain countries such as India are sparse and the burden of CKD is difficult to evaluate [9]. CKD is defined as estimated glomerular filtration rate (eGFR) < 60 ml/min/1.73m^2^ or urinary albumin-to-creatinine ratio (UACR) > 30 mg/g [1]. CKD and UACR are associated with higher all-cause mortality, also in older people [10]. A large collaborative meta-analysis including longitudinal data on more than 100,000 participants found that eGFR below 75 ml/min/1.73m^2^ and an UACR above 5 mg/g started to be associated with all-cause and cardiovascular mortality, independently of each other [11]. CKD patients suffer from high cardiovascular morbidity and mortality, which is mainly attributable to cardiovascular events before reaching end-stage renal disease (ESRD) [12-15].

The kidney is a key organ for the balance of many endogenous and exogenous compounds including drugs. Hence, the kidney is responsible for the excretion of numerous drugs and their metabolites. Some substances need to be actively maintained in the body (*e.g*., glucose, amino acids, *etc*.), while others need to be eliminated (*e.g*. urea, uric acid, exogenous compounds, *etc*.). When managing these various processes, the kidney may be damaged by the action of nephrotoxic substances (either endogenous or exogenous).

Several areas firmly link pharmacogenomics to the kidney as listed in Table **1**. We herein focus on the *third* context, namely the role of proteins involved in the metabolism and transport of drugs in renal function and blood pressure control to select the top three pharmaco-genomic applications to better understand renal patho-physiology in cardiovascular medicine. This review does not cover the use of pharmacogenomics in the field of renal transplantation as this area has been extensively covered in recent years [16-20]. Similarly, we do not explore the link between pharmacogenomics and acute renal failure.

There is large inter-individual variability in drug response [21]. Such variability has been shown to be heritable [22, 23]. It is likely that the inter-individual variability in response to other xenobiotics and to endogenous compounds is similarly large and also heritable. Selected genetic polymorphisms located within genes encoding drug-metabolizing enzymes (*e.g*., *CYP3A5*) or drug transporters (*e.g*., *ABCB1*) are considered to be functional. The strength of the association between genetic variability and function of the corresponding protein greatly varies across pharmacogenes. Genetic variants of the *CYP1A2* gene, for instance, show little association with CYP1A2 enzymatic activity [24], whereas *CYP3A5* genotype is an excellent predictor of CYP3A5 phenotype [25].

According to the Pharmacogenomics Knowledge Database [26, 27], 44 genes are classified as being very important pharmacogenes (VIP). In addition to the classical hypertension and renal function candidate gene *ACE*, recent evidence suggests that several of these genes may play a role in the physiological control of renal function and blood pressure, even in the absence of drug treatment [28-31]. The VIP genes we selected in this paper belong to four categories: a) phase I enzymes (*CYP1A2* and *CYP3A5*), b) transporters (*ABCB1*), c) nuclear receptors (VDR and PXR) and d) others (*ACE*, *MTHFR*). These genes are highly interconnected as illustrated in Fig. (**1**), which synthesize the crossroads between selected nuclear receptors, drug metabolizing enzymes and transporters as well as other pharmacogenes for their effects on renal function and diseases, in part *via *renal sodium handling.

The kidney plays a major role in blood pressure control and hypertension is considered as an important risk factor for nephropathy. As a consequence, these VIP genes represent important candidates for CKD and ESRD. In this review, we would like to highlight the complex interplay between inter-individual variability in response to drug treatment, inter-individual variability in the metabolism and transport of endogenous substances as well as inter-individual variability in the response to environmental factors (*e.g*., dietary salt intake) for their effects on renal function and blood pressure. The link between hypertension and kidney disease is complex because it is bidirectional. Reduced renal function is associated with raised blood pressure and hypertension is a risk factor for nephropathy. From a genetics perspective, this complexity is illustrated by the limited overlap between blood pressure and renal function loci (and candidate genes) identified so far in large scale genome-wide association studies. For instance, in the recently published genome-wide meta-analysis of the International Consortium on Blood Pressure genetics, a genetic score based on 29 variants associated with blood pressure was associated with stroke and heart disease, but not with renal function or CKD [32].

Blood pressure levels aggregate in families and are heritable. This suggests that genes play a role in blood pressure control. As a result of intra-individual blood pressure variability, heritability estimates are usually stronger and more significant when multiple measurements are averaged, such as is the case for ambulatory blood pressure monitoring [33]. When the average of only two measurements is taken, the blood pressure phenotype is noisier and very large sample sizes are needed to detect small genetic effect sizes. One way to ensure that a specific genetic association with blood pressure is not a false positive is to replicate results across independent samples as was done in large-scale genome-wide meta-analyses. There is no consensus on how to define salt-sensitivity of blood pressure [34], but one usually considers that a person is salt-sensitive whenever her/his blood pressure increase by 5% to 10% in response to a large increase in dietary salt intake (*e.g*., 200 mmol Na/d versus 20 mmol Na/d) [34].

This paper brings together a critical synthesis of recent evidence linking selected VIP genes, namely *ABCB1*, *ACE*, *CYP1A2*, *CYP3A5*, *MTHFR, PXR* and *VDR*, to (1) renal function, (2) blood pressure and (3) salt sensitivity focusing on, though not restricting to, results in humans (Table **2**). Notably, we highlight and present the rationale for three leading applications that we consider as important and actionable therapeutic and preventive focus areas in renal pharmacogenomics: 1) ACE inhibitors, as a *confirmed* application, 2) VDR agonists, as a *promising* application, and 3) moderate dietary salt intake, as a *suggested* novel application. In the course of this discussion, we underscore the potential role of gene-environment interactions, discuss the implications of these findings to treat and prevent hypertension and CKD and bring up new ideas for research in the coming decade to accelerate this under-studied and yet critical subfield of pharmacogenomics on the path to personalized medicine.

## 2 SELECTED VIP GENES: BLOOD PRESSURE; SALT-SENSITIVITY AND RENAL FUNCTION

### 2.1 Phase I Enzymes

#### 2.1.1 CYP1A2 Gene 

The *CYP1A2* gene lies on chromosome 15q24.1, shares a 5’-flanking region with *CYP1A1* and features seven exons [35]. *CYP1A2* encodes a member of the cytochrome P450 superfamily enzyme, the CYP1A2 enzyme. CYP1A2 is responsible for about 13% of the cytochrome P450 activity of the liver and is involved in the metabolism of several commonly used drugs (*e.g*., caffeine, clozapine, flutamide, lidocaine, melatonin, mexiletine, *etc*) and endogenous compounds [35]. Expression of *CYP1A2* is primarily regulated by the aromatic hydrocarbon receptor (AhR) [35]. There is a great inter-individual CYP1A2 variability [36]. CYP1A2 activity also shows high interethnic variability which can be attributed, in part, to differences in genetic variants and their frequencies [37] and possibly also to different lifestyle and environmental conditions across ethnic groups*. *CYP1A2 is an inducible enzyme whose activity is modified by various factors. On one hand, factors such as cigarette smoking, coffee consumption, intake of charcoal-grilled meat, omeprazole and carbamazepine induce CYP1A2. On the other hand, fluvoxamine and oral contraceptives (OCs) inhibit CYP1A2 enzyme activity [35].

In addition to environmental factors, the inter-individual CYP1A2 variability could be due to genetic factors [38]. For example, the CYP1A2*1C allele, identified in the 5’-flanking region of the gene, could lead to direct decreased CYP1A2 activity [39]. CYP1A2*1F allele has been suggested to confer a higher inducibility of CYP1A2 by smoking [40]. However, in a study with sequencing data on the entire *CYP1A1*/*CYP1A2* locus, no single SNP or haplotype could unequivocally predict CYP1A2 activity [24], and none of the currently identified *CYP1A2* polymorphisms seems to explain the large inter-individual variability in CYP1A2 activity.

##### CYP1A2 and Kidney Function

There are data suggesting a relationship between the *CYP1A2 *gene, CYP1A2 enzymatic activity and kidney function. These data come notably from studies showing that, in CKD patients, metabolic clearance of drugs, and particularly drugs metabolized by cytochrome P450, are decreased [41]. Decreased clearance could be due to a decrease in P450 protein expression, which in turn, would be secondary to a reduced gene expression. To better determine which *CYP-P450* gene and isoform were down-regulated in CKD, renal failure was induced in animal model and the effects on protein expression, gene expression, and P450 isoform activity investigated [42]. Compared to control animals, CKD mice had a reduced level of CYP1A2 protein expression (- 56%, p<0.05). In addition, a significant decrease in mRNA levels of CYP1A2 protein was also observed in CKD mice, which could explain the decrease in protein expression. While this study and others [41] confirm that the decreased clearance is secondary to a decrease in P450 gene and protein expression, association of *CYP1A2* with kidney function itself has not been shown. *CYP1A2* was not among the loci uncovered in association with GFR, CKD, or albuminuria in GWAS studies [43-46]. At the time being, it seems that *CYP1A2* gene does not have a major direct influence on kidney function.

##### CYP1A2 and Blood Pressure

Contrary to kidney function, evidence on an independent association of *CYP1A2* with blood pressure is available. GWAS have identified the region nearby *CYP1A2* as being robustly associated with blood pressure and hypertension [47]. Compared to A allele, the rs1378942 C variant was associated with higher diastolic blood pressure (beta coefficient: 0.41 mm Hg per copy of the C allele, standard error [SE]: 0.06, p value 2 x 10^-12^) and with a 10% increased risk of hypertension (Odds ratio [OR], 1.10, 95%CI 1.07-1.12, P value=2 x 10^-14^) [47]. A joint meta-analysis of the CHARGE and Global BPGen studies identified a region adjacent to *CKS-ULK3-CYP1A2* (rs6495122) as a genome-wide significant loci for diastolic blood pressure (beta coefficient: 0.40, SE: 0.06, p value=1.8 x 10^-10^) [48]. This signal was also identified in GWAS conducted in African-American (rs6495122, p value=3.7 x 10^-4^) [49] and Japanese (rs1378942, p=6 x 10^-3^) [50] populations, but not in two other GWAS conducted in east Asians [51] and Han Chinese population [52]. This suggests that the *CKS-ULK3-CYP1A2 *locus does confer some susceptibility to hypertension, but probably not across all race/ethnicities.

*CYP1A2* polymorphisms have been previously associated with disease susceptibility such as cancers, porphyria cutanea tarda, spontaneous abortion [53], but never with hypertension until these recent GWAS. The mechanisms by which *CYP1A2* influences blood pressure remain to be determined. Given the large (>100) number of substrates including drugs (*e.g*. clozapine, caffeine), procarcinogens (*e.g*. aflatoxin b1), and endogenous substrates (*e.g*. steroids) reported for CYP1A2, the mechanisms could largely be mediated by CYP1A2 substrates.

Although limited compared to other *CYP*-family genes, data on *CYP1A2* pharmacogenomics are available and the topic has been reviewed elsewhere [54]. The main attention has been paid to antipsychotic drugs, theophylline, and melatonin [38]. With respect to antihypertensive drugs, the focus has been made on CYP2C9, which metabolizes several antihypertensive angiotensin II receptor antagonists, such as losartan, irbesartan, candesartan and valsartan. For example, the *CYP2C9* genotype has been shown to influence losartan metabolism and to predict the blood pressure response to irbesartan [55]. Concerning *CYP1A2*, the results from a nutrigenomic study provide additional mechanistic hypotheses on the relationship between *CYP1A2* and blood pressure. In a matched case-control study (N=4,028) of subjects living in Costa Rica, an increased risk of myocardial infarction with increasing coffee consumption has been reported among carriers of the *CYP1A2* *C* variant [56]. For carriers of the slow metabolizing *1F allele, the adjusted OR (95%CI) of nonfatal myocardial infarction associated with consuming < 1, 1, 2-3, and 4 or more cups of coffee per day were 1.00 (reference), 0.99 (0.69-1.44), 1.36 (1.01-1.83), and 1.64 (1.14-2.34), respectively. Corresponding ORs for individuals with the rapid *1A/*1A genotype were 1.00, 0.75 (0.51-1.12), 0.78 (0.56-1.09), and 0.99 (0.66-1.48) (P value for gene x coffee interaction=0.04). The increased risk has been attributed to a prolonged presence of caffeine in the circulation among slow metabolizers due to lower enzyme activity. Through a comprehensive search of the human genome involving over 40,000 participants, Cornelis *et al*. recently discovered *AHR* and *CYP1A1/CYP1A2* locus associated with habitual caffeine consumption [57]. Caffeine is the most widely consumed stimulant in the world, with an estimated 80-90% of adults reporting regular consumption of caffeine-containing beverages [58]. The effects of coffee or caffeine intake on health in general and on cardiovascular disease, in particular, have been assessed with inconclusive results [59]. On the short term (*i.e*. less than 3 months), regular coffee or caffeine intake increases blood pressure [60], but a tolerance to the acute cardiovascular effects of caffeine has been described. There is no clear evidence that regular caffeine intake on the long run increases the incidence of hypertension [61, 62]. It is therefore not clear whether a phenomenon similar to the increased risk of myocardial infarction associated with increasing coffee consumption among carriers of specific *CYP1A2* variant could explain, at least in part, the increase risk of hypertension associated with *CYP1A2* variants in some GWAS studies. Finally, as the *CKS-ULK3-CYP1A2* locus contains many other genes than *CYP1A2*, further work is needed to know which gene is causally associated with blood pressure and hypertension.

In summary, *CYP1A2* encodes the CYP1A2 enzyme, which has a large number of endogenous and exogenous substrates. There is a great inter-individual CYP1A2 variability, which seems to be mainly due to environmental factors, but signals near the *CYP1A2* gene have been identified and replicated in at least three GWAS. The putative mechanism of association between *CYP1A2* and blood pressure is currently unknown. Possible mechanisms include an action mediated by CYP1A2 substrates, notably caffeine, and genes located near the signal.

#### 2.1.2 CYP3A5 Gene 

Members of the human cytochrome P450 (CYP) 3A subfamily (mainly CYP3A4 and CYP3A5) play an important role in drug metabolism [63]. It is currently considered that CYP3A activity accounts for about 50% of hepatic CYP activity, attributable mainly to the CYP3A4 enzyme [64, 65]. Yet, CYP3A4 and CYP3A5 have very similar substrate specificities [65]. The *CYP3A5* gene is located on chromosome 7q22.1, in a gene cluster containing also *CYP3A4*, *CYP3A43* and *CYP3A7*. The existing linkage disequilibrium between the *CYP3A4* and *CYP3A5* loci may make it difficult to clearly identify which is the true underlying causal variant. In this review, we concentrate on the *CYP3A5* gene because of its association with blood pressure and renal function [28, 30, 66] and because it is the predominant CYP3A form in the kidney [67]. The *CYP3A5* gene displays high nucleotide diversity in Caucasians [68] and appears to be under high selective pressure in non-Africans [69]. CYP3A5 is expressed in the kidney, liver, intestinal tract, adrenal gland and lung [64, 67, 70].

Unlike what is observed for *CYP3A4*, there is a strong association between *CYP3A5* genetic variants and CYP3A5 activity.****The *CYP3A5* rs776746 variant (6986G>A), located in intron 3, which determines the CYP3A5*3 (6986A) allele, influences mRNA splicing and leads to a premature stop codon as well as greatly reduced CYP3A5 enzymatic activity [25]. The wild type CYP3A5*1 allele (6986G) is associated with high CYP3A5 activity and only people carrying the CYP3A5*1 allele express large amounts of CYP3A5 [25]. Alleles other, and less frequent, than CYP3A5*3 are associated with reduced CYP3A5 activity, such as the CYP3A5*6 (which lead to a splicing defect) and CYP3A5*7 (which leads to a frameshift with premature termination of translation) alleles [64]. The frequency of the CYP3A5*3 allele, which varies substantially across ethnic groups and geographical locations, is significantly correlated with distance from the equator [69]. The frequency of the CYP3A5*1 allele ranges from 36% to 94% in people of African descent, from 5% to 15% in Caucasians and from 23% to 40% in Asians [64, 69, 71-73]. The regulation of *CYP3A5* expression seems to be influenced by multiple nuclear receptors, such as the glucocorticoid receptor (GR) [74, 75], pregnane X receptor (PXR) [76] and constitutive androstane receptor-beta (CAR) [76]. Smoking was found to reduce *CYP3A5* expression *in vitro *[74].

##### CYP3A5, Blood Pressure and Hypertension

Many candidate gene studies have explored the association between *CYP3A5* variants and blood pressure or hypertension in humans [29]. So far, results have been inconsistent. Furthermore, the *CYP3A5* locus did not come out in GWAS for blood pressure and hypertension [47, 48, 51, 77]. The CYP3A5*1 allele was associated with higher blood pressure in some studies [67, 72, 78, 79], with lower blood pressure in other studies [80, 81] or was not associated with blood pressure in other studies [72, 79, 81, 82]. In a meta-analysis including 2799 cases and 6794 controls from ten studies, Xi *et al.* found the *CYP3A5* rs776746 polymorphism to be significantly associated with blood pressure in Caucasians only [66]. Caucasian carriers of the CYP3A5*1 allele had lower systolic blood pressure (-1.3 mm Hg, 95% confidence interval:-2.401;-0.242) than non-carriers. In a case-control study including 250 pregnant women with and 250 without hypertension, *CYP3A5* rs776746 was not associated with hypertension in pregnancy [83]. In a large population-based study (Rotterdam study) [84], a genetic risk score composed of three “salt-sensitivity” variants (*CYP3A5* rs776746; *ADD1* G460T and *GNB3* rs2301339) was associated with blood pressure in type 2 diabetic patients only.

There are several mechanisms by which CYP3A5 may influence blood pressure, if it does. CYP3A5 converts cortisol to 6 beta-hydroxycortisol in the kidney [85, 86]. In rats, CYP3A activity correlates with systolic blood pressure [86] and inhibitors of CYP3A activity decrease the level of 6 beta-hydroxycortisol and blood pressure [85]. In humans, some evidence suggest that CYP3A5*1 carriers may have increased proximal tubular sodium reabsortion [28, 67, 72]. Hence, CYP3A5 could influence blood pressure by sodium and water retention in the kidney. The *CYP3A5* gene may therefore represent a salt-sensitivity gene, as detailed below. Fromm *et al. *[80] found people carrying the CYP3A5*3/*3 genotype to have lower serum aldosterone values. Eap *at al* [30] found that the CYP3A5*1 allele tended to be associated with higher plasma aldosterone levels. It therefore appears that *CYP3A5 *variants may be associated with activity of the renin-angiotensin-aldosterone system, which is a key regulator of blood pressure.

##### CYP3A5 Pharmacogenomics 

As already mentioned, CYP3A4 and CYP3A5 have similar substrate specificity [65]. Among CYP3A substrates, we can mention the following drugs prescribed in cardiovascular conditions*:*amlodipine, atorvastatine, celiprolol, cerivastatin, digoxin, diltiazem, enalapril, felodipine, losartan, lovastatin, nicardipine. Nifedipine, pravastatin and verapamil [65, 87]. So far the majority of pharmacogenetic studies on *CYP3A5* concentrated on tacrolimus, midazolam and ciclosporin [64]. *CYP3A5* genotypes clearly influence the pharmacokinetics of the immunosuppressant tacrolimus *in vivo* [64]. A limited number of studies have analyzed the role of *CYP3A5* variants on the response to CYP3A substrates used to treat cardiovascular conditions and most of these studies were small-sized, which increases the risk of false positive results and limits their external validity. *CYP3A5* variants appear to influence the pharmacokinetics of statins [88]. Carriers of CYP3A5*1 may have diminished pharmacological effect of verapamil [89], a nondihydropyridine calcium channel blocker that can be used to treat hypertension. Eap *et al. *[30] found the combined action of *CYP3A5 *and *ABCB1* variants to be associated with altered response to lisinopril, an ACE inhibitor. In the randomized African-American Study of Kidney Disease and Hypertension Trial, *CYP3A5* rs776746, unlike *CYP3A4* variants, was not associated with blood pressure response to amlodipine [90]. In 40 healthy Korean men [91], carriers of the CYP3A5*3/*3 genotype had lower plasma concentration of amlodipine than CYP3A5*1 carriers but the blood pressure response to amlodipine was similar*.*

##### CYP3A5, Salt-sensitivity and Dietary Salt Intake

Ho *et al. *[72] found CYP3A5*1 carriers to be more salt-sensitive than non-carriers. The frequency of the CYP3A5*3 allele is significantly correlated with distance from the equator and has similar geographic distribution than the *AGT M235T*, a variant associated with blood pressure and hypertension [69]. According to Thompson *et al. *[69], these results suggest that genetic variants influencing blood pressure sensitivity to salt underwent selective pressure that shaped their geographical distribution. In 375 people of African descent, CYP3A5*1 carriers tended to have higher age-related increase in blood pressure and proximal tubular sodium reabsorption than non-carriers [28], which suggests that the CYP3A5*1 allele is associated with high blood pressure sensitivity to salt. In the same group of people, whenever urinary sodium excretion was high, the CYP3A5*1 allele was associated with higher blood pressure in the absence of the *ABCB1 3435T* allele [30]. In this latter study [30], there was a significant gene-gene-environment (*CYP3A5*-*ABCB1*-salt intake) interaction. Similarly, in 238 Japanese men [92], the association of the *CYP3A5* rs776746 with blood pressure differed by salt intake level (*i.e*., gene-by-environment interaction). In 6777 participants to the PREVEND study [81], systolic blood pressure and pulse pressure were significantly lower in carriers of CYP3A5*1 with effect modification by sodium intake*. *In this latter study, men carrying the CYP3A5*1 allele had lower urinary sodium excretion than non-carriers and a similar, although not significant, trend was observed in women [81].

Plasma peak concentration following oral administration of quinidine, a CYP3A substrate, was found to be lower under high dietary salt intake than under low salt intake [93]. Similarly, high dietary salt intake was associated with lower plasma peak concentration of orally administered verapamil [94]. No such effects were observed after i.v. administration [93, 94]. These data suggest that dietary salt intake may influence the intestinal disposition of drugs metabolized by CYP3A. In rats, high-salt diet increased *Cyp3a3* expression and protein levels in the liver and intestine, but not in the kidney [95], which suggests that the effect of salt intake on gene expression and enzymatic activity is tissue-specific. Surprisingly little has been published during the past 10 years about the role (or the absence of role) of dietary salt intake on drug disposition. Kosuge *et al. *[96] recently observed *in vitro* that the transcription of *CYP3A5* is influenced by the osmotic environment in various cell lines, a process driven by Nuclear factor of activated T cells 5 (NFAT5), also called tonicity-responsive enhancer binding protein. Considering the key role of salt in driving osmotic forces, it is conceivable that high dietary salt intake may influence intestinal drug disposition. More generally, tonicity changes within the intestinal lumen following the intake of selected food items may well influence CYP3A expression [96] and therefore intestinal disposition of drugs and endogenous compounds that are CYP3A substrates.

##### CYP3A5 and Renal Function

There is little data regarding the association between *CYP3A5* variant and renal function. Givens *et al*. [67] found higher creatinine clearance CYP3A5*1 carriers in a study including 25 African Americans. Bochud *et al. *[28] found that CYP3A5*1 carriers had lower GFR estimated using inulin clearance than non-carriers in a study including 375 people of African descent. Lieb *et al.* [82] found no association of CYP3A5*1 with estimated GFR in women, but a weak association with lower estimated GFR in men. Fromm *et al. *[80] found no association of CYP3A5*1 with GFR in 115 young Caucasian men. Further studies are clearly needed to clarify the role of *CYP3A5* variants in renal function in humans.

In summary, there is strong correlation between *CYP3A5* variants and CYP3A5 activity. Yet, there are large inter-individual and inter-ethnic variations in CYP3A5*1 allele frequency. There is some evidence that the *CYP3A5* gene is associated with blood pressure control and some indirect evidence that it may influence salt-sensitivity in humans. Further studies are needed to confirm and further explore these relationships. Similarly, the association of *CYP3A5* variants with renal function reported in a few studies needs further confirmation. Among the putative mechanisms for the association with blood pressure is the convertion, by CYP3A5, of cortisol into 6 beta-hydroxycortisol in the kidney. It would be of major interest to also clarify the putative role of CYP3A5 activity on intestinal drug disposition following various dietary salt intake levels.

### 2.2 Transporters

#### 2.2.1 ABCB1 Gene

The *ABCB1* gene encodes the P-glycoprotein (Pgp), which belongs to the superfamily of human ABC transporters. The gene contains 29 exons and is located on chromosome 7q21.12. *ABCB1* is also known as the multi-drug resistance (MDR) gene because its overexpression in cancer cells leads to resistance to various anticancer drugs. Several *ABCB1* genetic variants have been shown to influence Pgp expression in humans, including the 3435 C>T and 2677 G>T variants. The 3435 C>T variant, although non-synonymous, influences mRNA stability [97] as well as Pgp substrate stability [98]. Pgp is expressed in many organs, including kidney, adrenal gland, liver, brain, testis and intestine. In the kidney, Pgp has been found in endothelial cells [99], on the apical surface of proximal tubular epithelial cells [100], in the mesangium, in Henle’s loop and collecting duct [101]. The regulation of Pgp expression seems to be influenced by multiple nuclear receptors, such as pregnane X receptor (PXR) [102], constitutive androstane receptor-beta (CAR) [103] and vitamin D receptor (VDR) [104-106].

Pgp is a very efficient efflux pump that transports many endogenous substrates (*e.g*. steroids, lipids, phospholipids and cytokines), drugs (*e.g*. digoxin, cyclosporine, tacrolimus, diltiazem, verapamil, *etc*), and other exogenous substrates out of the cells [107]. Among Pgp substrates, we will highlight the links with endogenous hormones that influence blood pressure, *i.e*. aldosterone and cortisol [108]. For a list of drugs that are substrates or modulators of Pgp, see the extensive review by Zhou [109]

##### ABCB1, Blood Pressure and Salt-sensitivity

Increasing evidence points toward a role of Pgp in the regulation of the renin-angiotensin-aldosterone system [108, 110-120], which is an important regulator of blood pressure and sodium handling by the kidney. Some reports suggest that Pgp is directly linked to endogenous components of the renin-angiotensin-aldosterone system. Pgp is probably involved in the transport of aldosterone in rodents and in humans [108, 110, 111]. In young healthy women, the *ABCB1* 3435C>T variant was associated with serum aldosterone level during the normal menstrual cycle [112]. The *ABCB1* 3435C>T was associated with the aldosterone and urinary sodium excretion responses to angiotensin II stimulation in young health men [118]. The *ABCB1* 3435C>T was associated with circulating endogenous ouabain levels, a hormone produced in the adrenal gland known to regulate, together with aldosterone, the activity of the sodium pump, a key player in blood pressure control [120].

Several reports point toward biological interactions between Pgp and drugs influencing the renin-angiotensin-aldosterone system. Losartan, an angiotensin-receptor blocker, is a Pgp substrate. Pgp appears to inhibit the efflux of aliskiren, a renin inhibitor used in the treatment of hypertension**,** in the small intestine [119]. Spironolactone, a non-specific aldosterone antagonist, up-regulates Pgp expression and efflux activity *in vitro* [113]. Telmisartan and the prodrug candesartan-cilexetil, angiotensin receptor type 1 blockers, were found to significantly inhibit Pgp activity [121, 122], which implies that important drug-drug inter-actions may occur when these drugs are combined with Pgp substrates. Studies in rats [123] and humans [124] found that the Pgp inhibitor cyclosporine A influences the renin-angiotensin-aldosterone system. Cyclosporine A belongs to the group of calcineurin inhibitors used to induce immuno-suppression after organ transplantation. Presence of the *ABCB1* variant haplotype 1236T/2677T/3435T in the donor was associated with long-term decrease in renal function in kidney transplant recipients receiving cyclosporin A [125]. By contrast, *ABCB1 *variants were not associated with renal function in 160 heart transplant recipients on calcineurin inhibitor treatment [126]. Finally, there is ample evidence suggesting that Pgp is involved in cyclosporine-induced post-transplantation nephrotoxicity [127-129], likely by influencing cyclosporine absorption [130]. Cyclosporine-induced post-transplantation nephrotoxicity is frequently associated with arterial hypertension.

Pgp plays a role in the transport of cortisol at the blood–brain barrier [131, 132]. Pgp may therefore influence local glucocorticoid activity in selected regions of the brain. Excess of glucocorticoids, whether of exogenous (*e.g*. glucocorticoid treatment) or endogenous (*e.g*. Cushing’s syndrome) origin, often leads to arterial hypertension, but the pathophysiological mechanisms are so far not well understood [133]. An excess of glucocorticoids may increase blood pressure *via *several mechanisms: (1) activation of the mineralocorticoid receptor and resulting enhanced renal sodium reabsorption, (2) activation of the renin-angiotensin-aldosterone system, (3) induction of sleep apnea and insulin resistance, (4) decreased vasodilation and (5) increased vasoconstriction [134].

Pgp not only has many substrates, but also many inhibitors and inducers [107, 135]. Numerous exogenous substances, including drugs, were found to inhibit (*e.g*. curcumin, ginsenosides, bergamottin from grapefruit juice, cyclosporin) or activate (*e.g*. clotrimoxazole, St John’s wort, some catechins from green tea) Pgp activity [107, 135] . Grapefruit juice was found to activate the transport of drugs by Pgp *in vitro* [136]. In rats, high salt diet substantially decreased *mdr1a *and* mdr1b* expressions and Mdr1 protein levels (these are the rat equivalents of *ABCB1* and Pgp in humans) in the kidney and liver, while an increase occurred in the intestine [95, 137]. This further emphasizes the potential for gene-environment, drug-environment and drug-drug interactions to modulate the effects of Pgp on blood pressure and renal function.

##### ABCB1 and Renal Function

Although humans express a single Pgp gene (*ABCB1* or *MDR1*), rodents share the function of *mdr1 *between two highly homologous genes, *mdr1a *and *mdr1b. *Single knockout (KO) mdrd1a (-/-) mice are viable and fertile, but they display increased neurotoxicity to ivermectin and vinblastine [138], but their renal function has not been determined [139]. Double Pgp KO mice [mdrd1a/mdr1b -1a/mdr1b (-/-)] have higher mean arterial pressure, proteinuria and 24-hour urine volume, reduced GFR and renal plasma flow compared to wild-type mice [139]. Furthermore these KO mice have higher lithium clearance, which suggests reduced proximal tubular sodium reabsorption, and show evidence of proximal tubular dysfunction [139]. By contrast KO mice were protected against ischemic renal injury [139]. Under normal conditions, mdrd1a/mdr1b (-/-) mice have higher water intake than wild-type mice and under low salt diet, KO mice display a rise in plasma aldosterone levels, unlike wild-type mice [139]. Mdrd1a/1b (-/-) mice have higher aldosterone activity in the plasma, brain and heart than wild-type mice, which suggests that Pgp is crucial in aldosterone disposition [114]. These animal data strongly suggest that Pgp plays a key role in blood pressure and renal function, in particular proximal tubular function.

In 290 people of African descent, the *2677T* and *3435T* *ABCB1* alleles were associated with higher GFR, measured using inulin clearance and higher effective renal plasma flow, measured using PAH clearance [31]. In the same study, we found *ABCB1* variants to be significantly associated with estimated GFR in a large population-based study in Caucasians [31]. These observational epidemiological data in humans suggest that the *ABCB1* gene is indeed a candidate gene for renal function in humans. Yet, this gene has not been identified in large scale genome-wide association studies (GWAS) on renal function [43, 140, 141], CKD [43, 140, 141] or microalbuminuria [44]. One possibility to explain this lack of replication is that these GWAS meta-analyses were underpowered to detect all relevant genetic variants with small effect sizes, another one is that gene-environment interactions may obscure the role of this gene when pooling results across studies and, finally, these [31] may have been false positive results. Furthermore, we found the *3435T* *ABCB1* and CYP3A5*1 variants to interact with dietary salt intake for their effect on ambulatory blood pressure [30]. Hence the association of the 3435T *ABCB1* variant with blood pressure could only be observed when taking CYP3A5*1 status and dietary salt intake into account. This may explain why the *ABCB1* gene has not been identified in GWAS meta-analyses for blood pressure and hypertension [47, 48, 77]. Here again, in the absence of replication, false positive results [30] cannot be excluded.

Cadmium, a common environmental pollutant, enhances the expression of *ABCB1* *in vitro* [142, 143]. Renal proximal tubular cells that overexpress Pgp are more resistant to the toxic effects of cadmium [144]. In humans, chronic exposure to cadmium leads to its accumulation in the kidney with subsequent kidney damage, in particular in the proximal tubules [45]. Another possible link between kidney damage and chronic exposure to cadmium is *via *blood vessel damage in the kidney. All layers of the wall of blood vessels (intima-media-adventitia) are a target for cadmium deposition [145]*. *Chronic exposure to cadmium has been associated with atherosclerosis in humans and in animals [146]. In the population-based NHANES study, high blood cadmium levels were associated with increased risk of albuminuria and reduced GFR [147]. However, according to Nawrot *et al.*, cadmium-induced kidney damage may not drive cadmium-related mortality [45]. By contrast, there is no clear association between 24-h urinary cadmium excretion, a marker of lifetime exposure to cadmium, and blood pressure or hypertension [45]. Further studies are needed to clarify the role of Pgp in cadmium-induced nephropathy.

In summary, variants within the *ABCB1* gene and its product, Pgp, may influence blood pressure and renal function in many different ways, not only *via *action on, and interaction with, endogenous substances (including components of the renin-angiotensin-system), but also *via *their effects on the transport of many drugs and other exogenous substrates or effect modifiers (including environmental pollutants and dietary factors). Disentangling the specific effects of Pgp substrates and effect modifiers, as well as their possible interactions, in complex living organisms such as mammals is a daunting task and much remains to be done to better understand how Pgp may regulate blood pressure and renal function. There is a clear lack of experimental studies in humans aiming at understanding the role of Pgp under physiological conditions as well as in response to selected stressors such as low and high salt intake. Also, we do not know if, and to what extent, Pgp may play a role in the progression toward CKD in humans.

### 2.3 Nuclear Receptors

#### 2.3.1 VDR Gene

The *VDR* gene is located on chromosome 12q13.1 and encodes the vitamin D receptor (VDR) [148]. VDR is expressed in most cells of mammals, but primarily in intestine, bone and kidney. VDR is widely expressed in the human kidney, namely in epithelial cells of the proximal and distal tubules, collecting duct and glomerulus [149]. VDR is a ligand-induced nuclear receptor that regulates the expression of over 900 genes throughout the genome [150, 151], among which *ABCB1 *[104-106], *CYP24A1 *[150], *CYP3A4 *[152], *CYP3A7 *[150], *FGF23 *[153], *SLC34A3 *[153] and *TRPV6 *[150, 154]. VDR is a protein that plays a crucial role in the biological responses to vitamin D, the synthesis of which is briefly presented below. However, VDR may also have vitamin-D independent actions triggered by low-affinity ligands such as lithocholic acid [152], curcumin and polyunsaturated fatty acids [155].

Vitamin D is derived from three sources: 1) sunlight –the main source–, 2) diet, and 3) dietary supplements [156]. The solar ultraviolet V (UVB) causes photolysis of the 7-dehydrocholesterol (provitamin 3) to previtamin D3 and then to vitamin D3 (cholecalciferol) in the plasma membrane of skin cells (PMID 12520530). Diet and dietary supplements are sources of vitamin D3 and vitamin D2 (ergocalciferol). Both vitamin D3 and D2 enter the blood circulation and are attracted to the vitamin D binding protein (VDbp). A first hydroxylation by CYP27A1 in the liver produces the 25-hydroxyvitamin D [25(OH)D], while a second hydroxylation (*i.e*., 1 alpha hydroxylation) by CYP27B1 in the kidney produces the 1,25-dihydroxyvitamin D [1,25(OH)2D3], which is the hormonally active form [157]. Extrarenal 1,25(OH)2D3 can also be produced and 1,25(OH)2D3 can act locally in the tissues where it is produced [158].

While some of 1,25(OH)2D3 actions take place directly in the cellular membranes without interaction with the cellular nucleus (*i.e*. vitamin D rapid response), 1,25(OH)2D3 mostly acts through binding specific intracellular receptors (*i.e*. vitamin D genomic responses) [159, 160]. The 1,25(OH)2D3 dissociates from the VDbp, enters the cell and interacts with the vitamin D receptor and activates it. The VDR-1,25(OH)2D3 complex translocates from the cytosol to the nucleus where it is joined by the Retinoid X Receptor (RXR), to form the vitamin D response elements (VDRE). The complex then binds to specific sequence in the promotor of reponsive genes and modulates the gene expression [160].

In the intestine, the VDR-1,25(OH)2D3 complex regulates genes that are involved in the absorption of calcium and phosphate, while the VDR-1,25(OH)2D3 complex regulates the parathyroid hormone synthesis in the parathyroid glands. But the VDR protein is widely distributed in humans (*e.g*., lung, colon, kidney) and VDR abundance and activity seems to play an important role in the individual responsiveness to 1,25(OH)2D3. Some of the VDR abundance and activity is determined by VDR polymorphisms [161].

The coding sequence of the *VDR* gene includes eight exons. Several polymorphisms have been identified but four of them are common and have often been studied (FokI, BsmI, TaqI, ApaI). FokI is located at the *VDR* start codon and affects the length of the VDR protein resulting in a change in VDR activity [161]. BsmI, TaqI, and ApaI polymorphisms are located in the UTR region of the *VDR* gene and are unlikely to change the VDR structure. However, these polymorphisms might alter transcriptional activity and mRNA degradation, and thus VDR abundance, which is an important mechanism for the modulation of cellular responsiveness to 1,25(OH)2D3.

##### Vitamin D, VDR, and Kidney Function

The kidney 1 alpha hydroxylation is decreased in kidney failure, and patients with ESRD typically suffered from 1,25(OH)2D3 vitamin D deficiency, while usually keeping a normal 25(OH)D level [162]. Vitamin D therapy is often prescribed to ESRD patients with secondary 1,25(OH)2D3 deficiency. The causal role of vitamin D deficiency on kidney failure has only been explored recently. In 2009, Ravani *et al*. showed that baseline low 25(OH)D levels were associated with an increase risk of ESRD [163] compared to patients with normal 25(OH)D levels ***.*** In a prospective cohort study of 9,000 hemodialysis patients, vitamin D therapy appeared to be associated with survival [164]. There are however no prospective, population–based studies of the association of 25(OH)D levels with change in kidney function or incidence of CKD other than ESRD. Experimental studies suggest that vitamin D levels can directly or indirectly prevent kidney failure. Kidney failure typically results from three kidney lesions: 1) tubulointerstitial fibrosis, 2) glomerulosclerosis, and 3) proteinuria [165]. The effects of paricalcitol (19-nor-1,25-hydoxy-vitamin D2), a synthetic vitamin D analogue, on tubulointerstitial lesions have been investigated in animal models. Compared with vehicle controls, paricalcitol significantly attenuated renal interstitial fibrosis [166] and both 1,25(OH)2D3 and oxacalcitriol (another vitamin D analogue) ameliorates glomerulosclerosis with reduction of type I and IV collagenes in antibody-induced glomerulonephritis [167]. The reduction of albuminuria [168-170] or proteinuria [171] in CKD patients have been reported in three randomized controlled trials comparing placebo to vitamin D analogues. Oral calcitriol treatment reduced proteinuria in patients with IgA nephropathy [172]. Yet, another mechanism by which vitamin D can presumably modify kidney function is through the suppression of the renin-angiotensin system (RAS). The renal RAS plays a major role in determining the rate of chronic renal progression [173]. In 1986, Resnick *et al*. already reported that serum level of 1,25(OH)2D3 was inversely associated with the plasma renin activity in normotensive and hypertensive subjects [174]. Since then, 1,25(OH)2D3 and paricalcitol were found to decrease angiotensinogen, renin, and renin receptor in animal models [175, 176]. This suggests that the beneficial effects of vitamin D analogues in chronic renal failure are due, in part, to down regulation of the RAS.

The role of *VDR* genetic variants on kidney function has been explored only recently and current data are mostly limited to ESRD. The influence of *VDR* polymorphism on ESRD was investigated 258 ESRD patients and 569 healthy controls [177]. A significant difference in the genotype frequencies of the ApaI, FokI and BsmI genotypes were found. In addition, the ApaI/TaqI/FokI/BsmI haplotype analysis revealed that subjects with a/t/F/b haplotype were at greater risk of ESRD (OR =11.0, 95%CI 1.38-87.7) [177]. This study confirmed the B allele to be the risk allele as being previously reported in a case-control study including 222 subjects with and without ESRD [178].

These results contrast with a previous report analyzing the influence of BsmI variants on PTH and 1,25(OH)2D3 in patient with different degrees of CKD before dialysis [179]. Most of the 248 patients included had moderate kidney failure (*i.e*., creatinine clearance 35-60 ml/min). In multivariate analyses, calcitriol levels were less reduced in the BB genotype and the progression of hyperparathyroidism was slower in patients with Bsm1 BB genotypes than in the other types [179].

Albuminuria serves as an important predictive factor for the progression of kidney disease and for the development of cardiovascular disease. The contribution of genetic variants, including *VDR*, to the development of albuminuria has been evaluated in 5321 participants from the second phase (1991-1994) of the Third National Health and Nutrition Examination Survey (NHANES III), a population-based and nationally representative survey of the United States [180]. Albuminuria was evaluated as logarithm-transformed albumin-to-creatinine ratio (ACR), as ACR ≥ 30 mg/g, and as ACR above sex-specific thresholds. *IL1B* (rs1143623) among Mexican Americans was significantly associated with increased sex-specific albuminuria. *IL1B* (rs1143623), *CRP* (rs1800947) and *NOS3* (rs2070744) were significantly associated with ACR ≥ 30 mg/g among Mexican Americans (P < 0.05). In contrast, no variants were found to be associated with albuminuria among non-Hispanic blacks after adjustment for multiple testing. The only variant among non-Hispanic whites significantly associated with any outcome was *TNF* rs1800750, which failed the test for Hardy-Weinberg proportions in this population. Among Mexican Americans, haplotypes within *ADRB2* (A-G), *IL4r* (C-A), and *VDR* (T-T) were associated with log(ACR) in crude genetic models only (P value <0.05). Age-sex adjusted models were only marginally associated with log(ACR).

Further information can be derived from the influence of *VDR* polymorphisms on CKD major risk factors; diabetes and hypertension (for hypertension, see below). BsmI *VDR* polymorphisms have been associated with diabetes and fasting glucose. Compared to men with BsmI Bb genotype (N=370) and bb genotype (N=245), carriers of BB genotype had higher levels of fasting glucose (5.61 vs 5.44 and 5.38 mmol/l, p value <0.001) in individuals recruited during routine medical qualification for flying duty in Germany [181]. These associations were only reported in gene carriers with low physical activity [181]. The association of BsmI *VDR* polymorphism with type 2 diabetes mellitus was analyzed in 293 patients considered at high risk for coronary artery disease [182]. The prevalence of type 2 diabetes mellitus was gradually dependent on the number of B alleles (BB 28%, Bb 13%, bb 8%, P value = 0.002). The odds ratio of having type 2 diabetes mellitus was 3.6-fold higher among patients with the BB genotype compared with patients with the bb genotypes [182]. The relationship between *VDR* BsmI and FokI polymorphisms and anthropometric and bio-chemical parameters describing metabolic syndrome were tested in 176 randomly selected men in Poland. The BsmI *VDR* polymorphism seems to influence BMI and waist circumference with BB carriers having higher BMI (29.0 vs 26.8, p=0.024), and waist circumference (101.8 vs 92.3, p value=0.014) compared with bb genotypes. Fok1 *VDR* polymorphism appears to affect insulin sensitivity with FF and Ff carriers having higher level of fasting insulin then ff genotpyes (12.3 vs 9.8 vs 6.3, p value=0.008) [183]. In the rancho Bernardo study, a US community-based study of unrelated older adults without known diabetes, the genotypes frequencies of ApaI, BsmI, and TaqI polymorphism did not differ between persons with and without diabetes. Fasting plasma glucose and prevalence of glucose intolerance were higher in nondiabetic persons with aa genotype compared with those with AA genotype. BsmI bb genotype was associated with insulin resistance in subjects without diabetes [184]. In a brief genetic report, an association between ApaI genotype and insulin secretion was reported in a study of 164 healthy Bangladeshi Asians at risk for type 2 diabetes; aa genotype was associated with a decrease insulin secretion compared to AA and Aa genotypes (68.5 vs 92.7 vs 146.8, p value=0.001) [185]. In a separate publication but within the same study population, TaqI was associated with insulin secretion [186].

The pharmacogenomics of vitamin D and kidney function have been largely unexplored so far. To the best of our knowledge, there is no study that explored the *VDR* genetic effect modification of the association between 1,25(OH)2D3 and kidney function. Nor could we find study on the pharmacogenomics of vitamin D analogues with respect to kidney function. The lack of pharmacogenomics information on renal-related *VDR *pharmacogenomics is surprising given the association between vitamin D deficiency and CKD, and given the high prevalence of vitamin D supplementation / food fortification in North America [187]. There are inter-individual differences in vitamin D optimisation and recent evidence of lack of response to oral vitamin D in subjects who harbor specific *VDR* variants. In postmenopausal women, *VDR* BmsI and TaqI polymorphisms were association with non response to vitamin D oral supplementation [188]. Given the ongoing debate on recommendations of daily allowance of vitamin D, notably for cardiovascular disease prevention, further pharmacogenomics and nutrigenomics data should be gathered including in patient with non ESRD CKD who might benefit from vitamin D.

##### Vitamin D, VDR, and Blood Pressure

Molecular, animal and human studies have established that vitamin D is associated with cardiovascular disease, including blood pressure. This topic has been reviewed elsewhere [189]. Three randomized controlled trials assessed the efficacy of vitamin D supplementation on blood pressure. Only one found a significant effect [190]. Compared with calcium alone (1,200 mg/day), vitamin D (800 IU/day) and calcium (1,200 mg/day) supplements resulted in 9.3% decreased systolic blood pressure (p = 0.02) in a 8-week trial including 148 women (mean age 74 years) with a 25(OH)D level < 50 nmol/l [190]. In 2008, Wang *et al. *[191] investigated the associations of vitamin D intake with the incidence of hypertension in a 10-year prospective cohort of 28,886 US women aged 45 or more years. Vitamin D intake was assessed from food frequency questionnaire. The risk of hypertension decreased in the higher quintiles of dietary vitamin D, even after adjustment for dietary calcium intake. This observation was reported for vitamin D intake from diet, not from supplements. Most of the large cross-sectional studies show a significant inverse association between 25(OH)D levels and blood pressure [189]. The number of prospective studies examining 25(OH)D levels and the incidence of hypertension or change in blood pressure are limited and results are inconsistent. Most studies were small, had suboptimal blood pressure measurement (*e.g*., single measure of blood pressure), or did not control for potential confounders such as PTH.

Molecular evidence revealed actions of 1,25(OH) 2 D on mechanisms related to blood pressure. These mechanisms include a direct inhibition of 1,25(OH) 2 D on the RAS and nuclear factor-kappa B (NF-kB) pathway. *VDR* is expressed in the juxtaglomerular apparatus and modulates renin synthesis. Mice in which *VDR* was abolished are hyperreninemic and present high blood pressure and cardiac hypertrophy [192]. By contrast, when *VDR* was over-expressed in the mouse juxtaglomerular apparatus, hypor-eninemia was noted [193]. NF-kB is a family of transcription factors that functions as a master regulator of immune response [194]. It regulates a wide range of genes involved in inflammation, proliferation and fibrogenesis and is known to have a key role in kidney disease [195]. Both the RAS and the NF-kB promote the production of profibrotic and pro-inflammatory factors, increase oxidative stress, and damage podocytes. In addition, vitamin D can regulate blood pressure through the prevention of secondary hyperparathyroidism, effect on vascular cells and endothelial function. Vitamin D could potentially contribute to arterial stiffening and hypertension [196]. Another mechanism by which VDR may influence blood pressure is *via *its role in sex steroid metabolism [197]. Sex hormones have been found to influence systemic and renal hemodynamic response to salt [198]. In *VDR* null mice, the expression and activity of aromatase (CYP19A1), a key hormone in estrogen biosynthesis, was reduced in ovary and testis and circulating levels of LH and FSH increased compared to wild-type mice [199]. *VDR* null mice presented uterine hypoplasia that resulted from a lack of estrogen synthesis that was not observed in vitamin D-deficient mice [200]. In human cells, vitamin D increased the expression of *CYP19A1 *[201] and the convertion of 17-b-estradiol to estrone [202], which suggests that VDR mediates estrogen synthesis in humans as well.

Associations of *VDR* polymorphisms with blood pressure have been explored. In a study including 76 patients of Indian descent and 201 patients of African descent, vitamin D deficiency was significantly associated with increased diastolic blood pressure and triglyceride levels, and reduced high-density lipoprotein cholesterol (P<0.05) [203]. Prevalence of vitamin D deficiency was decreased in patients carrying the f allele of FokI (OR: 0.52, 95%CI 0.30–0.90, P=0.02) and the aa genotype of ApaI (OR: 0.46, 95%CI 0.21–0.99, P=0.05). BsmI and TaqI SNPs were not associated with vitamin D deficiency [203].

The prevalence of the *VDR* BsmI polymorphism and its association with anthropometric and biochemical features of metabolic syndrome, including high blood pressure, were examined in 351 randomly selected healthy postmenopausal women. BsmI genotypes were not associated with blood pressure nor with other anthropometric or metabolic parameters except LDL cholesterol level, which was higher among BB carriers [204]. The relationship between *VDR* gene polymorphisms (Bsm-I, Apa-I and Fok-I) and target organ damage with essential hypertension was explored in 74 patients without types 2 diabetes mellitus or impaired glucose tolerance and severe obesity [205]. No significant difference was detected in biochemistry results and physical examination between groups for Bsm-I and Apa-I *VDR* gene polymorphisms. A negative correlation was present between vitamin D levels and day-time interval and early morning average by the measurement of 24-hour ambulatory blood pressure in the non-FF group. In addition, the degree and presence of retinopathy were significantly higher in the non-FF group when compared to the FF group (p = 0.025, p = 0.018, respectively) [205]. An association of *VDR* polymorphisms with 25-hydroxyvitamin D on blood pressure in apparently healthy subjects was also reported for BsmI [206]. In a cross-sectional study of 590 subjects and conducted in Spain, systolic blood pressure was higher in men with bb genotype than in the other genotypes (P < 0.006). This association was not seen among women [206].

##### VDR Gene and Salt-Sensitivity

No study has assessed the association between *VDR* genetic variants and salt-sensitivity. However, a recently published study found 25-hydroxyvitamin D to be associated with plasma renin activity and with salt-sensitivity in patients with low-renin hypertension in 223 Caucasian hypertensive patients [207].

In summary, variants within the *VDR* gene and its product, VDR, may influence renal function and blood pressure. Randomized controlled trials provide convincing evidence that VDR agonists confer renoprotection in humans [168-170, 208]. Associations of vitamin D with renal function, and *VDR* gene with renal function, have been demonstrated, but there is a clear lack of data on the potential effect modification of *VDR* variants on the association between vitamin D and renal function. The existence of dietary (non-vitamin D) ligands of VDR suggests that gene-environment (*i.e*. *VDR*-diet) interactions may impact on the biologic actions of VDR, but this has not been studied so far. *VDR* variants have been associated with blood pressure (including 24-hour ambulatory blood pressure) and target organ damage. Evidence of associations between *VDR* variants and diabetes, a major CKD risk factor, and biochemical features of the metabolic syndrome have been reported. There is little data on *VDR* pharmacogenomics, mostly limited to bone disease.

#### 2.3.2 PXR Gene 

The Pregnane X Receptor (*PXR*) gene belongs to the 1I family of nuclear receptors, together with *VDR *and *CAR*. PXR is a ligand-regulated transcription factor that modulates the expression of many genes, including genes encoding for drug metabolizing enzymes and transporters [209]. *PXR* is located on chromosome 13q12-13.3 and includes 9 exons. Seven alternative transcripts have been described [210]. More than 200 *PXR* variants have been described so far, including several variants that alter the aminoacid sequence of PXR [211-217]. *PXR* is highly expressed in human liver, intestine, lung and kidney [218].

PXR binds the enhancer elements located in the promoters of many genes, such as *CYP2B9*, *CYP2C8, CYP2C9*, *CYP3A4, CYP3A7 *and *ABCB1*, thereby influencing the expression of these genes [219]. Other gene targets of PXR are *SULT2A*, involved in bile acid detoxification [220], *UGT1A1*, *OATP2*, *GSTA1*, *GSTA2* and *MRP2, *involved in bilirubin clearance pathway [221].

Single nucleotide polymorphisms (SNPs) in the *PXR* gene could influence PXR activity as they have been shown to influence the expression of *PXR* gene targets [214], such as *CYP3A4 *[212, 216, 217, 222] and *ABCB1* [217]. As the enzymes and transporters encoded by PXR target genes are involved in the control of many endogenous systems, PXR is expected to influence many normal physiological and disease processes even in the absence of drug treatment [209]. PXR is implicated in bile detoxification and cholestasis, bilirubin detoxification, adrenal steroidogenesis, lipid metabolism, bone homeostasis, retinoic acid metabolism and inflammation [209].

PXR activity is modulated by a vast array of agonists or ligands, such as drugs (*e.g*. digoxin, spironolactone, nifedipine, rifampicine) and xenobiotics (*e.g*. polychlorinated biphenyls [PCBs], DDT, St John’s wort, sulforaphane) [211], but also endogenous compounds (pregnane, progestrones, corticosterones, testosterone, lithocholic acids, 17-a ethinylestradiol) [223, 224] and vitamins K2 and E [225, 226]. In fact, PXR was named pregnane X receptor because of its ability to be activitated by C21 steroids [224]. Among PXR agonists are several endocrine-disrupting chemicals (*e.g*. 17-a ethinylestradiol, DDT, PBCs, dioxins), *i.e*. compounds that may affect human reproductive function and fertility. Also, recent experimental studies in rats found dietary salt intake to modulate the expression of *PXR* differentially in the liver, kidney and intestine [95]. Unlike nuclear receptors such as estrogen receptors, PXR has high structural flexibility so that it can bind structurally very diverse ligands [211].

Increasing evidence suggests that there is an intensive crosstalk between nuclear receptors of the NR1I subfamily (*i.e*. PXR, VDR and CAR) [227]. These receptors share the ability to enhance the metabolism and elimination of toxic endogenous and exogenous substances [227]. Also, these receptors share multiple transcriptional targets (*e.g*. *CYP2B6*, *CYP3A4*, *ABCB1*, *ABCB2*, *UGTA1*, *CYP24A1, SUL2A1*) [220, 227, 228].

##### PXR, Blood Pressure and Salt-sensitivity

So far, no study has shown any evidence of a direct link between the *PXR* gene and blood pressure in humans. Yet, the role of PXR in adrenal steroidogenesis [209] is particularly relevant to blood pressure control. More generally, PXR is considered to influence glucocorticoid and mineralocorticoid homeostasis [197]. In mice, activation of PXR led to increased circulating levels of corticosterone and aldosterone [229]. These mice had adrenal cortex hyper-trophy and lacked glucocorticoid circadian rhythm [229]. The action of *PXR* on the mineralocorticoid pathway makes it an ideal hypertension candidate gene. Also, there is some evidence the PXR may influence vascular tone during pregnancy in PXR knockout mice [230]. *PXR-/-* mice were resistant to the vascular relaxation due to 5b-dihydropro-gesterone treatment [230]. The authors of this latter study hypothesize that this PXR effect could be mediated *via *PXR-activation of CYP epoxygenases [230]. The role of PXR in steroid hormone homeostasis has been shown to also act *via *induction, in the liver, of the phase II UGT1A enzyme, which is involves in the elimination of steroids, such as corticosterone, cortisol and cortisone, from the body [228]. Also, rifampicine, a PXR ligand, was found to lead to high urinary free cortisol excretion in humans [231]. However, the *PXR* locus did not come out in GWAS for blood pressure and hypertension [47, 48, 51, 77]. Also, we did not find any study providing a direct link between the *PXR* gene and blood pressure salt-sensitivity.

##### PXR and Renal Function

We are not aware that any study ever found evidence for a direct link between the *PXR* gene and renal function in humans. The *PXR *locus was not uncovered as associated with CKD or other renal traits in GWAS [43, 44, 141]. Yet, recent evidence suggests that nuclear receptors in general, and PXR in particular, may play a role in renal diseases *via *their actions on lipid and energy metabolisms and inflammation [208].

In summary, although *PXR* is not currently considered as a gene involved in blood pressure or renal function, its role in controlling the expression of genes such as *ABCB1* and *CYP3A5*, its involvement in steroid hormone metabolism, its actions on lipid and energy metabolism and inflammation as well as its interaction with VDR, all point toward PXR as being a putative important player in kidney diseases. The role of PXR as a xenobiotic and endobiotic sensor and its ability to bind to a large array of ligands, together with its numerous transcriptional gene targets, suggests that PXR may mediate complex gene-environment, drug-environment and drug-drug interactions with important consequences on human health, including kidney function.

### 2.4 Other Pharmacogenes

#### 2.4.1 ACE Gene

The angiotensin converting enzyme (*ACE*) gene encodes ACE, an enzyme involved in the renin-angiotensin-aldosterone system (RAAS) and in the kinin-kallikrien pathway that play a key role in blood pressure control. ACE is the target of ACE inhibitors, a family of drugs used to treat cardiovascular and renal diseases, including hypertension [232]. Renin converts angiotensinogen into angiotensin I, which is in turn converted by ACE into angiotensin II, the major effector of the RAAS. Among other functions, angiotensin II influences sympathetic tone, vasoconstriction and aldosterone secretion, which leads to sodium retention by the kidney.

The *ACE* gene is located on chromosome 17q23.3 and contains 26 exons. Alternative splicing leads two ACE isoforms. The most extensively studied *ACE* genetic variant is the 287-bp Alu-repeat sequence insertion/deletion polymorphism located in intron 16 (*ACE* *I/D*) [233], with more than 4,000 publications during the past 20 years [234]. There is a high inter-individual variability in circulating ACE levels. The *ACE I/D* polymorphism explains about half of the phenotypic variance of circulating ACE levels [233], which highlights its functional importance. The *D* allele is associated with higher circulating ACE levels [233]. Yet, there is a controversy regarding the precise nature and location of the functional *ACE* polymorphism(s) [235]. Other *ACE* variants have been described and the genetic diversity of particularly high in people of African descent [235-237]. However, most studies have analyzed the *ACE I/D* polymorphism and we will therefore concentrate on this variant. The *ACE* gene is expressed in many tissues including the kidney.

##### ACE and Blood Pressure/Hypertension

Similarly to what is found for most blood pressure candidate genes in humans, results of associations between *ACE* variants and blood pressure and/or hypertension have been inconsistent, as illustrated by an absence of overall association in two meta-analyses [238, 239]. A major issue with the majority of candidate gene studies is their small sample size and hence low power to detect a small effect. By contrast, a meta-analysis including data on 11,000 Han Chinese found the *ACE DD* genotype to be associated with higher risk of hypertension when compared to the *ACE II* genotype (OR[95%CI] = 1.61 [1.32-1.96]), but there was a high degree of heterogeneity across studies [240]. The *ACE* locus did not come out in large genome-wide meta-analyses [47, 48, 51, 77]. In a recently published analysis of 30 gene regions encoding antihypertensive drug targets including data on 87,000 people, the *ACE* *I/D* (rs4305) variant was found to be modestly associated with hypertension [241]. Results regarding hypertension-related cardiovascular complications are also not consistent. In a meta-analysis including 6638 subjects from 28 studies [242], the *ACE D* allele was associated with higher left ventricular hypertrophy in never treated hypertensive patients, whereas no association was found in treated patients. The *ACE I/D* polymorphism was not associated with the risk of myocardial infarction, ischemic heart disease or stroke in one meta-analysis [239], whereas in another meta-analysis [238], the *ACE D* allele was associated higher risk of coronary heart disease, myocardial infarction, stroke and diabetic nephropathy. In a meta-analysis including 43,733 coronary artery disease cases and 82,606 controls [243], the *ACE D* allele was associated with higher risk of coronary artery disease (OR[95%CI]=1.25 [1.16-1.35]). In the latter study [243], there was a high degree of heterogeneity, in particular across ethnic groups. In this meta-analysis, the results were stronger in men [243]. Several studies have identified an effect modification of sex for the effect of the D allele on the risk of coronary artery disease [243]. In a meta-analysis including 7,500 Han Chinese [244], the *ACE DD* genotype was associated with higher risk of stroke (OR[95%CI]=1.91 1.56-2.34). In a meta-analysis involving 1121 patients with preeclampsia and 1361 controls from 11 studies [245], the *ACE DD* genotype was associated with increased risk of preeclampsia (OR95%CI=1.51 1.17-1.94).

##### ACE and Renal Function

A meta-analysis of 42 studies including 13,000 participants (published between 1994 and 2010) reported an association between the *ACE I/D* polymorphism and diabetic nephropathy [246]. The *ACE D* allele was associated with increased risk of diabetic nephropathy (OR[95%CI]=1.24 [1.12-1.37]) [246]. This was also true for subgroup analysis in type 1 nephropathy, type 2 nephropathy, ESRD and proteinuria [246]. When stratifying by ethnic group, this association was only observed in Asians and not in Caucasians [246]. The *ACE I/D* polymorphism therefore appears to be a useful test to predict the progression of type 1 or type 2 diabetic nephropathy [247].

The role of the *ACE I/D* polymorphism in non-diabetic renal disease is less well established [247]. In 2847 participants to the MESA study, the *ACE I/D* polymorphism was not associated with renal function [248]. The *ACE* locus did not come out from large scale genome-wide meta-analyses of renal function or CKD [44, 140, 141]. In a meta-analysis, the *ACE I/D* polymorphism was not associated with ESRD or risk of hypertension in patients with autosomal dominant polycystic kidney disease [249]. The *ACE D* allele was also associated with increased risk of minimal change nephritic syndrome in Asians (OR [95%CI]=1.38[1.07-1.79]) [>50]. In a meta ->1 studies [250], the *ACE DD* genotype was associated with susceptibility to, and progression of, IgA nephropathy with stronger associations in Asians than in Caucasians.

##### ACE and Salt-sensitivity

Only few studies have analyzed the association between the *ACE I/D* polymorphism and blood pressure salt-sensitivity. Hypertensive patients carrying the II genotype had higher ambulatory blood pressure increase under high salt intake than patients carrying the *DD* genotype [251]. In this study [251], the *ACE I* allele was therefore associated with higher salt-sensitivity than the D allele. This is in line with one study including 66 hypertensive patients in Japan [252] and with a study including 71 hypertensive patients in Spain [253], whereas other Japanese studies including 104 [254] and 188 [255] hypertensive patients found no such association. Although large scale studies are needed to confirm this, it seems that the I allele of the *ACE I/D* polymorphism confers blood pressure sensitivity to salt intake.

##### ACE and Pharmacogenetics

In an overview published in 2006, Arnett *et al*. identified 11 studies having analyzed the role of the *ACE I/D* polymorphism on blood pressure response to ACE inhibitor treatment, but the results were inconsistent [256]. In particular, the *ACE I/D* polymorphism was not associated with blood pressure response to ACE inhibitor (perindopril) treatment, or with the long-term risk of stroke and cardiac events, in 5,688 participants to the PROGRESS trial [257]. In a study including 208 Finnish hypertensive men, the *ACE I/D* polymorphism did not influence blood pressure response to treatment [258]. In the large scale GenHAT study including data on 37,000 people, the *ACE I/D* polymorphism did not significantly predict fatal and nonfatal coronary heart disease, myocardial infarction, stroke or all-cause mortality [259]. Similary, in 8907 patients with stable coronary artery disease from the EUROPA trial [260], the *ACE I/D* polymorphism was not associated with ACE inhibitor treatment benefit (*i.e*. no association with cardiovascular morbidity and mortality during follow-up). By contrast, in this same study, ACE inhibitor treatment benefit was associated with variants in the AT1 receptor and BK1 receptor genes [260]. These results suggest that the *ACE* *I/D* polymorphism is unlikely to be clinically relevant in predicting response to antihypertensive treatment or risk of cardiovascular disease in the absence of renal dysfunction.

The situation is quite different in the presence of renal dysfunction or in the presence of high salt intake [247]. Multiple lines of evidence suggest that the *ACE DD* genotype is associated with reduced renal benefit from ACE inhibitor treatment in patients with type 1 or type 2 diabetic nephropathy [247, 261]. In 1435 type 2 diabetic patients with nephropathy, who participated to the RENAAL study, the *ACE D* allele was associated with poor renal prognosis in the placebo group, whereas no such association was found in the losartan group, which suggests that losartan was able to correct the poor prognosis associated with the *ACE D* allele [262]. Patients carrying the *DD* genotype respond less well to ACE inhibitor treatment under conditions of high salt diet [263]. In 27 healthy subjects, angiotensin I infusion led to higher blood pressure, renal vascular resistance, and aldosterone levels in those carrying the *DD* genotype than in those carrying the *ID* and *II* genotypes under liberal sodium intake, but not under low sodium intake [264]. Similar results were obtained in type 1 diabetic patients [265]. These results are compatible with a gene-environment interactions between the *ACE I/D* polymorphism and dietary sodium intake for their effects on blood pressure and renal function.

In summary, ACE is an enzyme involved in the renin-angiotensin-aldosterone and kinin-kallikrien pathways involved in blood pressure control. ACE inhibitors are used to treat cardiovascular and renal diseases, including hypertension. The high inter-individual variability in circulating ACE levels is explained, in large part, by the *ACE I/D* polymorphism. The *D* allele of the *ACE I/D* polymorphism is clearly associated with increased risk of diabetic nephro-pathy. By contrast, the associations of the *ACE I/D* polymorphism with hypertension and cardiovascular disease have been inconsistent. The *ACE I/D* polymorphism is a useful test to predict the renoprotective effect of ACE inhibitor or angiotensin receptor blocker treatment in patients with kidney disease [247]. There is currently no evidence to support a role of the *ACE I/D* polymorphism in predicting future risk of cardiovascular events or blood pressure response to ACE inhibitors in the absence of renal dysfunction.

#### 2.4.2 MTHFR Gene

* MTHFR* is located on chromosome 1p36.22 and encodes the methylenetetrahydrofolate reductase. This enzyme is a key enzyme for folate homeostasis and catalyzes the conversion of 5,10-methylentetrahydrofolate to 5-methyltetrahydrofolate, the substrate for the conversion of homocysteine to methionine [266]. The latter is involved in the methylation of DNA and proteins. *MTHFR *includes 11 exons [267] and several (>60) polymorphisms have been identified. Some of them are nonsynonymous polymorphisms and alter the protein product. For example, C677T (rs1801133) and A1298C (rs1801131) are associated with decreased MTHFR activity and increased homocysteine levels [268].

* MTHFR* genetic variations cause MTHFR deficiency, neural tube defects and some evidence suggests an association with cancer [269]. Severe deficiency in MTHFR causes elevated homocysteine concentrations, an atherothrombotic sulfur amino acid associated with increased risk of cardiovascular disease [270]. Meta-analyses on the associations between *MTHFR* genotype and coronary heart disease have provided conflicting results [271]. These analyses allow testing a causal role of elevated homocysteine concentrations while avoiding bias from reverse causation given the principle of mendelian randomization.

##### MTHFR, Homocysteine and Kidney Function

CKD patients and to a major extend patients on dialysis suffered from elevated homocysteine [272], which may explain a large proportion of the attributable mortality of CKD [273]. *MTHFR* polymorphisms have been associated with an increased risk of ESRD [274] and increased risk of cardiovascular risk in ESRD [275].

Data on the association between *MTHFR* and CKD patients at an earlier stage of ESRD are also available. *MTHFR* variants have been associated with accelerated chronic decline in renal function. The influence of *MTHFR* variants C677T and A1298C on progressive loss of kidney function has been analyzed in 821 African-Americans with hypertensive nephrosclerosis from the longitudinal National Institute of Diabetes and Digestive and Kidney Diseases African-American Study of Kidney Disease and Hypertension (AASK) Trial [276]. In this study, the A1298C variant predicted the rate of GFR decline. A1298/A1298 major allele homozygosity resulted lower decline of GFR. Subjects with A1298/A1298 had a decline of 0.034 mL/min/1.73 m2/week versus 0.048 and 0.042 mL/min/1.73 m2/week in heterozygotes and 1298C/1298C subjects, respectively [276]. This result was replicated in an independent follow-up study (San Diego VAHC study). C677T alone did not show an individual effect. The relationship of *MTHFR* gene polymorphisms with the presence and the severity of renal disease was investigated in 100 patients with CKD and 120 healthy controls Tunisians. C677T and A1298C polymorphisms were not associated with the presence of non-ESRD renal disease [277]. In a substudy of 677 patients with ESRD using dialysis (N=213) or advanced CKD (N=464) in the Homocysteinemia in Kidney and End-Stage Renal Disease [HOST] trial, the C677T genotype frequencies, but not A1298C, differed between patients with ESRD and those with advanced CKD [278]. Frequencies of the C677T CC, CT, and TT genotypes were 62%, 32%, and 7% in participants with ESRD and 54%, 35%, and 11% in participants with advanced CKD, respectively (P =0.03). This study also examined the mortality associated with *MTHFR* genotypes. In patients with ESRD with the mutant C677T TT genotype, the adjusted hazard ratio for mortality was 2.27 (95%CI 1.07-4.84; P = 0.03). The relationship was not significant in patients with advanced CKD. The overall relationship between the A1298C polymorphism and mortality was significant neither in patients with ESRD nor in patients with advanced CKD [278]. Studies conducted in other populations (*e.g*., Cyprus [279], Poland [280]) reported association between *MTHFR* variants and the risk of CKD. However, the *MTHFR* region was not uncovered as associated with CKD or other renal traits in recent GWAS [43, 44, 141].

Some of the mechanisms by which *MTHFR* mutations influence kidney function are possibly mediated by homocysteine. Elevated levels of homocysteine are associated with increased risk of thrombosis and *MTHFR* genetic variants are associated with inherited thrombophilia states [281]. Patients with inherited thrombophilia experience chronic thrombotic events including within the renal vasculature, which can result in gradual and progressive decline in renal function. The association of *MTHFR* C677T polymorphism with renal vascular sclerosis was compared between patients whose renal biopsies showed vascular disease (diabetic patients, hypertensive patients, diabetic and hypertensive patients, smokers, and idiopathic renal disease) and patients without vascular sclerosis at the biopsy [281]. In 34 patients with diabetes, hypertension, or both, there was no significant difference compared to the controls. However, in 17 patients with idiopathic vascular disease group, *MTHFR* C677T mutant polymorphism was associated with vascular sclerosis (29.4% in patients with idiopathic vascular disease versus 4% in controls, p value=0.03). These results suggest that patients without hypertension and diabetes but with idiopathic renal vascular disease might be predisposed to vascular scarring by thrombophilic gene mutations [281]. The atherothrombotic propriety of homocysteine potentially mediates the increased risk of vascular sclerosis and kidney failure. Some reports have indeed linked higher homocysteine levels to a greater decline of GFR [282], while others did not [283, 284] and the ultimate origin and pathophysiology for elevated homocysteine levels in kidney failure are still unclear [285].

The comparison of the relationship between *MTHFR*, homocysteine and kidney function across different settings can also be confounded by other environmental factors such as folic acid intake and fortification policy. *MTHFR* C677T is especially associated with moderate elevated homocysteine concentration in setting of marginal folate intake [286].

##### MTHFR, Homocysteine and Blood Pressure

Contrary to some of the other pharmacogenes discussed in this review, associations between *MTHFR* and blood pressure were already suggested before associations from GWAS on blood pressure and hypertension were available. For example, in 2004, a meta-analysis of studies comparing women with and without hypertension in pregnancy for the *MTHFR* C677T suggested that the T allele increases the risk of severe diastolic hypertension during pregnancy [287]. Nevertheless, important insights emerged from the GWAS. The *MTHFR* region has been uncovered as a loci associated with blood pressure or hypertension in multiple GWAS. A GWAS was conducted in 18,738 Caucasian women who participated in the Women’s Health Study cohort study and were free of hypertension at baseline. blood pressure progression at 48 months and incident hypertension during the entire follow-up according to the different genotypes were assessed [288]. *MTHFR* rs1801133 had a significant association with blood pressure progression. The OR for blood pressure progression (95%CI) for *MTHFR* rs1801133 (minor allele T) was 1.05 (1.00–1.10), but this association nor the association with incident hypertension remain significant after adjustment for multiple testing [288].

Analyses from the GlobalBPGen and CHARGE consortia data showed an association between systolic or diastolic blood pressure and variant (rs17367504) near the MTHFR gene (P=2 x 10^-13^) in the *MTHFR/CLCN6/NPPB* locus. Carriers of the G allele at this polymorphism were less likely to have hypertension compared to carriers of the A allele (OR 0.89, 95%CI 0.86-0.93, p value 2 x 10^-9^). The rs17367504 is located in an intron of the *MTHFR* gene but in a region with many plausible candidate genes, including *MTHFR*, *CLCN6*, *NPPA*, *NPPB* and *AGTRAP *[47]. Signal near the *MTHFR* gene was also uncovered in a recent GWAS conducted in Chinese Hans, but the association was limited to diastolic blood pressure [52]. Significant association with systolic blood pressure was replicated for *MTHFR* rs17367504 (beta coefficient: 0.65, p value= 0.03) among Japanese subjects (N=1526) [289].

The *MTHFR* region was also identified in the first large-scale (N=2020) experiment using the new Illumina HumanCVD BeadChip (50K IBC array) in relation to mean 24-hour systolic and diastolic blood pressure (GRAPHIC Study) [290]. A strong association between rs13306560 polymorphism in the promoter region of *MTHFR* and *CLCN6* and mean 24-hour diastolic blood pressure was found. Each minor allele of rs13306560 was associated with 2.6 mm Hg lower mean 24-hour diastolic blood pressure (P=1.2×10^−8^). Interestingly, this study also showed that a majority of the candidates previously associated with clinical measurements of blood pressure were not or only weakly associated with mean 24-hour blood pressure. The investigators stressed that mean 24-hour blood pressure has a higher heritability than clinic measurements and is probably more informative in gene discovery [290]. Unfortunately, the number of studies with data on both high-throughput genetic and 24-hour blood pressure information is currently too limited to replicate such analysis on a sufficient large scale.

While this study further suggests that *MTHFR* gene is a causal gene for hypertension, it is worth noting that some recent candidate gene studies and GWAS did not replicate the association. For example, *MTHFR* gene polymorphisms C677T and A1298C were not risk factors for hypertension in a recent candidate gene study conducted among 150 Turkish participants [291] and this signal was absent in a GWAS of Korean cohorts [292].

The influence of *MTHFR* polymorphisms on blood pressure and hypertension could again be mediated, at least in part, by homocysteine. Homocysteine levels have been associated with hypertension and the different mechanisms leading to hyperhomocysteinemia-associated hypertension have recently been reviewed [293]. Mechanisms include 1) activation of metalloproteinases with reduction of vascular elastance, 2) reduction of H2S production, a strong anti-oxidant and vasorelaxation factor, and 3) alteration of the physiological balance between the NO/endothelin system, which is responsible for vasodilation/vasoconstriction and remodelling processes of the vasculature. There is however some evidence that mild elevated homocysteine can not solely explain the hypertensive risk associated to *MTHFR* genotype. In a case-control study of 458 participants, an increased hypertensive risk was observed in *MTHFR* C677T TT carriers men with homocysteine level higher than 15 micromol/L (OR=2.78; 95%CI 1.05-7.3, p value=0.03) but not in those non-TT carriers men with homocysteine level higher than 15 micromol/L (p value=0.33) [294].

The pharmacogenomics of *MTHFR* in general has been reviewed elsewhere [266]. Below, we discuss the pharmacogenomics of *MTHFR* related to blood pressure. We identified studies that did investigate the modification of the association between benazepril treatment (an ACE inhibitor) and blood pressure by *MTHFR* C677T. In 2005, Jiang S *et al*. investigated in Chinese hypertensive patients whether short-term blood pressure response to benazepril, was modulated by haplotypes re-constructed from both C677T and A1298C polymorphisms in *MTHFR* gene [295]. A total of 410 hypertensive patients recruited from 344 nuclear families were treated orally with benazepril at a daily dosage of 10 mg for 15 consecutive days. Blood pressure was measured at baseline and on the 16th day of treatment. The individuals carrying one copy of haplotype 677C-1298C had significantly lower diastolic and systolic blood pressure response to benazepril treatment (beta coefficient=-1.9, p= 0.003 and beta coefficient =-2.9, p =0.043, respectively), in comparison to those without this haplotype. In 2011, the same group conducted a similar study, yet without haplotype analysis, but in a larger Chinese population (N=823). *MTHFR* C677T was associated with baseline systolic blood pressure (beta coefficient=2.84, P=0.0096) and baseline diastolic blood pressure (beta coefficient=2.19, P=0.0008). *MTHFR* C677T was independently associated with increased diastolic blood pressure response (baseline minus post-treatment) to benazepril treatment (beta coefficient =1.58, P=0.038), but not with systolic blood pressure response [296]. Thus, *MTHFR* C677T polymorphisms may modify some of the blood pressure response to benazepril treatment.

Vitamin B12, B6, and B2 (riboflavin) are the main nutritional determinants of homocysteine. Intervention studies based on vitamin B supplementation have generally reported no effect on blood pressure. As Wilson *et al*. discussed elsewhere, studies have rarely considered the *MTHFR* C677T polymorphisms [297]. One study did examine the effect of riboflavin (1.6 mg/day for 16 weeks) on blood pressure by *MTHFR* C677T polymorphism in 181 premature cardiovascular disease patients [298]. Among patients taking one or more antihypertensive drugs at recruitment, the target blood pressure (<140/90 mmHg) was achieved in only 37% patients with the TT genotype compared with 59% with the CT and 64% with the CC genotype (P < 0.001). Riboflavin intervention reduced mean blood pressure specifically in those with the TT genotype (from 144/87 to 131/80 mmHg; P < 0.05 systolic; P < 0.05 diastolic), with no response observed in the other genotype groups [298]. Williams *et al*. conducted an intervention study where 41 asymptomatic men with normal or high-normal ambulatory blood pressure were randomized to receive in a placebo-control, cross over study, 3 weeks of 5 mg folic acid/day or placebo. The investigators found a similar blood pressure response (decrease) to folate supplementation across *MTHFR* C677T genotypes [299]. It is worth noting that *MTHFR* genotypes have also been associated with risk of dyslipidemia and diabetes [300-302].

##### MTHRF and Salt-sensitivity

We could not identify studies addressing salt sensitivity and *MTHFR*. We suggest therefore that this is an area in need of further research investment. MTHFR is a key enzyme in folate and homocysteine metabolism. Selected* MTHFR* genetic variants cause MTHFR deficiency and are associated with neural tube defects and inherited thrombophilia states. Patients with impaired renal function have elevated homocysteine concentrations, and *MTHFR* polymorphisms have been associated with the risk of developing ESRD. In recent GWAS, the *MTHFR* locus was associated with blood pressure and/or hypertension, but not with renal function or CKD. As other plausible nearby blood pressure candidate genes exist (*e.g*., *NPPA,* *NPPB*) further studies are needed to determine whether *MTHFR* is indeed the causal gene. Homocysteine seems to explain only partially the associations between *MTHFR* polymorphisms and kidney function and blood pressure/hypertension. Some studies showed that *MTHFR* variants significantly modified the blood pressure response to antihypertensive drugs.

## CONCLUSIONS AND OUTLOOK

### Top Three Pharmacogenomic and Personalized Medicine Applications in Renal Pharmacogenomics: A Strategic Agenda

Pharmacogenes have been mostly studied for their effects on drug metabolism, safety and efficacy. Less is known on the roles of pharmacogenes in disease susceptibility or kidney pathophysiology. More specifically, we consider that the role of pharmacogenes for endogenous substrates in relation to renal function and blood pressure needs more in-depth investigation. While the genome-wide meta-analyses (GWAS) have not identified the VIP pharmacogenes as being robustly associated with renal function, these initial explorations do not necessarily address the entire genetic variability of renal function or blood pressure (so-called “missing heritability”). CKD is a growing public health burden worldwide and a better understanding of its pathophysiology is urgently needed, as are new therapeutic interventions. As illustrated in this paper, a substantial number of important pharmacogenes are associated with blood pressure and hypertension in humans. With the exception of VDR, the link with renal function is less clear, but this has been under-studied so far. Further studies are needed in this area because 1) the kidney is the major organ responsible for blood pressure control, and 2) renal function is an important determinant of the clearance of many drugs and other xenobiotics. This knowledge is important to better assess the role of pharmacogenes in health in general and to individualize treatment in particular.

In our view, ACE inhibitors represent the first pharmacogenomic application at the nexus of renal pathophysiology and cardiovascular medicine. As detailed in this paper, ACE inhibitor treatment is clearly renoprotective. Furthermore, the *ACE I/D* polymorphism is a useful test to predict the progression of type 1 or type 2 diabetic nephropathy [247] and also to predict benefit from ACE inhibitor treatment.

**Fig. (1):**
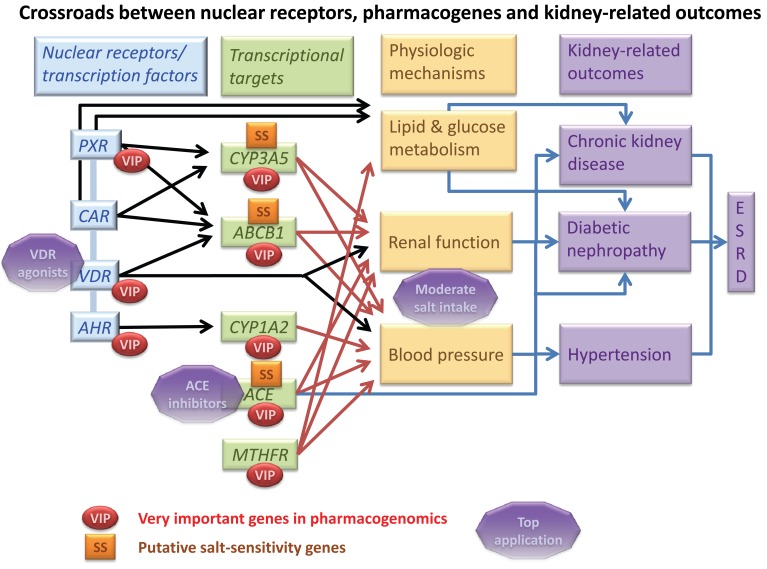
**Top three pharmacogenomics applications** at the nexus of renal pathophysiology and cardiovascular medicine, namely *ACE inhibitor treatment*, *VDR antagonists* and *moderate dietary salt intake*. Arrows represent the direct links as described in the scientific literature. The light blue band across *PXR*, *CAR*, *VDR* and *AHR* illustrates the tight interplay that exists among these genes. VIP = very important pharmacogenes; SS = genes that are involved in the blood pressure response to salt intake with various levels of evidence.

Among the selected pharmacogenes, two are nuclear receptors, namely VDR and PXR. The role of nuclear receptors in renal diseases has been recently highlighted with an emphasis on carbohydrate metabolism, lipid metabolism, immune response and inflammation[208]. We here add a new putative link between nuclear receptors and renal diseases, namely *via *blood pressure control and renal sodium handling (Fig. **1**). Lipid and drug metabolisms are also tightly interconnected *via *shared regulation by nuclear receptors such as PXR, CAR, VDR and GR [303]. PXR was originally described as a xenobiotic sensor, but it is now being increasingly recognized as an *endobiotic sensor* with roles in modulating inflammation as well as lipid, energy and vitamin D metabolisms [211]. We here put forward the hypothesis that PXR may also play a role in blood pressure control and renal sodium handling *via *its action on the transcription of the *ABCB1* and *CYP3A5 *genes. For the other nuclear receptor, VDR, the link with blood pressure and renal function, in particular, is clear, as previously mentioned. We therefore choose VDR agonists, such as vitamin D analogs, as our second pharmacogenomic application at the nexus of renal pathophysiology and cardiovascular medicine.

Several dietary factors may influence the expression and activity of pharmacogenes, among which grapefruit juice and herbal extracts such as St John’s wort stand out [95]. Dietary salt has been found to modulate quinidine and verapamil disposition, but only in case of oral administration [93, 94], which suggests that the influence of salt intake on gene expression is tissue-specific [95]. Recent data in mice showed that dietary salt intake influence the expression of several pharmacogenes, namely *CYP3A* genes, *ABCB1* and *PXR, *and that this effect is tissue specific [95]. More studies, including human studies, are needed to explore the role of dietary salt intake on the expression and action of pharmacogenes. The kidney is responsible for salt homeostasis. Although large inter-individual differences in the blood pressure response to salt intake exist, selected subgroups appear to be more salt-sensitive than others. In particular, high salt intake is known to increase blood pressure in elderly people, people of African descent and patients with hypertension, obesity or CKD. A large body of evidence suggests that reducing salt intake lowers blood pressure and reduces risk of cardiovascular disease [304-306]. Low salt intake is expected to be of particular benefit to patients with hypertension or reduced kidney function. So far, data on the genetic determinants of blood pressure sensitivity to salt are limited and results have been inconsistent [34, 307]. Among the reasons to explain these discrepancies, we can mention the fact that currently used protocols substantially vary across studies (*e.g*., one-week low salt versus one-week high salt, response to i.v. NaCl infusion, response to an i.v. diuretic dose, *etc*). Furthermore, as these research protocols are quite demanding, sample sizes have usually been very small, with rarely more than 100 participants, so that studies have been underpowered to detect small effect sizes. We are therefore lacking a salt-sensitivity test suitable for large scale population-based studies. Baring these limitations in mind, several pharmacogenes are currently considered as being salt-sensitivity genes (Fig. **1**). Yet, we know little on the role of dietary salt intake on the expression of these pharmacogenes. Furthermore, little has been done to explore the role of salt intake on the pharmacokinetics and pharmacodynamics of drugs in general and, of drugs used to treat essential arterial hypertension and renal dysfunction in particular. If salt intake plays an important role, this may have clinical consequences in adapting drug doses to achieve better treatment safety and efficiency, while maintaining optimal preventive strategy to reduce overall cardiovascular risk. We therefore choose ‘reduced dietary salt-intake’ as our third pharmacogenomic application at the nexus of renal pathophysiology and cardiovascular medicine for its potential to reduce cardiovascular risk in salt-sensitive individuals.

As previously suggested [308], we support that idea that randomized controlled trials should systematically include in-depth genotyping of participants, at least for important pharmacogenes to allow exploring inter-individual variability in drug response as well as gene-gene interactions in large samples.

As some of the pharmacogenes also act as xenobiotic sensors, multiple interactions between genes, drugs as well as other xenobiotics and dietary factors are expected. This underscores the need for large-scale international consortia to decipher the complex interplay between xenobiotics, endobiotics and pharmacogenes on kidney function and diseases in humans.

## Figures and Tables

**Table 1.** Interface Between Pharmacogenomics and the Kidney

Context for Research	Focus	Significance
1. Drugs used to treat renal pathologies acting on a protein encoded by a pharmacogene	Drugs	Inter-individual variability in drug response
2. Nephrotoxic drugs	Drugs	Inter-individual variability in nephrotoxicity
3. Proteins involved in the metabolism and transport of drugs and renal function and blood pressure	Endogenous compounds	Inter-individual differences in the progression of chronic kidney disease (even in the absence of drug treatment)
4. Interactions between endogenous and exogenous compounds	Endogenous and exogenous (drugs + others) compounds	Large inter-individual variability in age-related renal function decline

**Table 2.** Selected VIP Pharmacogenomics Genes: Renal Function, Blood Pressure and Salt-sensitivity

Gene	Top Three Applications	Level of Evidence Gene Top Three Applications Linking the Gene to:		
		Renal Function	Blood Pressure	Salt-sensitivity
*ABCB1*	Reduced salt intake	+	+	+
*ACE *	ACE inhibitors, Reduced salt intake	++	++	+
*CYP1A2*		_	+	_
*CYP3A5*	Reduced salt intake	+	+	+
*MTHFR*		+	+	_
*PXR *		_	(+)	_
*VDR *	VDR agonists	+	+	_

- No evidence; (+) Indirect evidence; + Direct evidence; ++ Strong evidence.

## ABBREVIATIONS

ABCB1: = ATP-binding cassette, subfamily B (MDR/TAP), member 1
ACE: = Angiotensin converting enzyme
ACEI: = Angiotensin converting enzyme inhibitors
CAR: = Constitutive androstane receptor
CKD: = Chronic kidney disease
CYP1A2: = Cytochrome P450, family 1, subfamily A, polypeptide 2
CYP3A5: = Cytochrome P450, family 3, subfamily A, polypeptide 5
ESRD: = End-stage renal disease
GFR: = Glomerular filtration rate
GWAS: = Genome-wide association studies
MTHFR: = Methylenetetrahydrofolate reductase (NAD(P)H)
PXR: = Pregnane X receptor
VDR: = Vitamin D receptor
VIP: = Very important pharmacogenes
